# Biomechanical Analysis Suggests Myosuit Reduces Knee Extensor Demand during Level and Incline Gait

**DOI:** 10.3390/s22166127

**Published:** 2022-08-16

**Authors:** Jaewook Kim, Yekwang Kim, Seonghyun Kang, Seung-Jong Kim

**Affiliations:** Department of Biomedical Engineering, Korea University College of Medicine, Seoul 02841, Korea

**Keywords:** gait, biomechanics, electromyography, joint kinematics, soft wearable robot, rehabilitation

## Abstract

An FDA-approved soft wearable robot, the Myosuit, which was designed to provide hip and knee extension torque has recently been commercialized. While studies have reported reductions in metabolic costs, increased gait speeds, and improvements in clinical test scores, a comprehensive analysis of electromyography (EMG) signals and joint kinematics is warranted because the recruitment of appropriate muscle groups during physiological movement patterns facilitates effective motor learning. Here, we compared the lower limb joint kinematics and EMG patterns while wearing the Myosuit with that of unassisted conditions when performing level overground and incline treadmill gait. The level overground gait sessions (seven healthy subjects) were performed at self-selected speeds and the incline treadmill gait sessions (four healthy subjects) were performed at 2, 3, 4, and 5 km/h. In order to evaluate how the user is assisted, we conducted a biomechanical analysis according to the three major gait tasks: weight acceptance (WA), single-limb support, and limb advancement. The results from the gait sessions suggest that Myosuit not only well preserves the users’ natural patterns, but more importantly reduce knee extensor demand during the WA phase for both level and incline gait.

## 1. Introduction

According to the World Health Organization and American College of Sports Medicine, adults should, at a minimum, engage in 150 min/week of moderate intensity physical activities in order to maintain good health [1,2]. The fulfilment of this requirement has been shown to increase cardio-pulmonary functions, metabolism, and mental health, and moreover decrease the risk of diseases such as cancer, cardiovascular diseases, and diabetes [1,3,4,5,6,7]. Even though copious amounts of evidence highlighting the importance of exercise have been presented, over 27.5% of the elderly population remain sedentary [2,8,9]. While the reasons for lethargic lifestyles vary from lack of motivation to severe chronic conditions, fatigue and decreased musculoskeletal capabilities are also considered to be significant contributors [10,11,12]. This presents severe concerns because it has been well documented that limited physical activity can lead to sarcopenia thus triggering a vicious cycle [10,11,13].

To avoid the deconditioning spiral, current strategies rely heavily on physical assistance provided via therapists and trainers during training sessions accommodated at specialized facilities or through at-home visitations. However, it is often found that constant attention of the specialist, or in some cases multiple specialists, is demanded. This presents added difficulties to the individual as limited human resources and added financial costs hinder frequent exercise [14,15,16,17]. While interventions and assistive devices such as slings and harness systems can be employed to somewhat alleviate this burden, the inherent restriction of movement may not be well suited for moderate- or high-intensity exercises.

High expectations of a new era of training and rehabilitation have been brought forth via advancements in robotics and more specifically soft wearable exosuits which offer more freedom of motion with less limb inertia compared to the hard exoskeleton type counterparts. Of particular interest is the FDA-approved Myosuit initially developed by the ETH Sensory-Motor Systems Lab and now commercially distributed via MyoSwiss [18,19]. The Myosuit actively assists the user by working in coordination with the knee and hip extensor muscles, which are the main contributors of bodyweight support, while also providing hip flexion assistance via passive elastic bands [18,19]. Moreover, the users’ gait phases are automatically detected such that assistance can be provided according to the relevant functions. Clinical studies with diverse participant groups suggest that the Myosuit is indeed well suitable for activity-based training by presenting evidence of metabolic cost reduction, joint movement perseveration, and improvements in various physical aptitude tests [20,21].

We would like to emphasize that precautions are warranted when assessing the viability of wearable robotics without addressing volitional muscle activity. This was made evident by the early versions of full-bodyweight-support gait rehabilitation robots which did not well facilitate desirable rehabilitative outcomes, despite providing adequate trajectory control and reduced metabolic costs, due to lack of patient participation [22,23,24]. Thus, it is imperative that the facilitation of volitional muscle activity is also established. Furthermore, the resulting joint movements should also be monitored in tandem with electromyography (EMG) signals such that the training sessions, which usually consist of high dosages of repetitive exercises, do not reinforce unnatural patterns [25,26].

This paper aims to serve as a preliminary investigation of changes in lower limb joint kinematics and EMG activity of healthy subjects while walking wearing the Myosuit. Our goal was to assess whether the Myosuit causes any non-physiological patterns during level walking and if the Myosuit can assist with more demanding tasks such as incline gait. We hypothesize that if the Myosuit does not induce large deviations in lower limb kinematics while reducing EMG levels, without altering the individual’s natural patterns, the Myosuit could be a viable solution for physiological training. In order to evaluate the data according to the major tasks involved with walking, we dissected the gait cycle into three distinct regions: weight acceptance (WA), single limb support (SS), and limb advancement (LA) [27,28,29]. The results suggest that the Myosuit well assists the subject by lowering the knee extensor demand during the WA phase of level and uphill walking without interfering with the physiological patterns.

## 2. Materials and Methods

### 2.1. Experimental Setup

**Figure 1:**
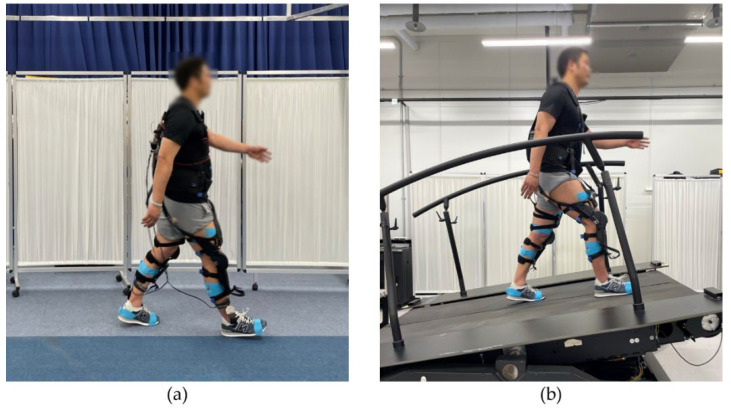
Gait sessions. A subject performing (**a**) level overground; (**b**) incline treadmill gait sessions while wearing the Myosuit.

This paper presents the results of two separate experiments brought together via a comprehensive analysis of joint angles and EMG patterns. Initially, we investigated whether significant adaptation strategies surfaced when walking while wearing the Myosuit by conducting overground gait sessions (Figure 1). The protocol was set up such that the biomechanical patterns of the Myosuit modes and assist levels could be evaluated. During each trial, we simultaneously monitored lower limb EMG activity and joint angles. As shown in Figure S1, the EMG signals were obtained via the soleus (SL), gastrocnemius medialis (GM), tibialis anterior (TA), biceps femoris (BF), rectus femoris (RF), and vastus medialis (VM), which have all been well documented to play crucial roles during gait. Additionally, the hip, knee, and ankle joint angles were estimated via 3D orientation information of the foot, shank, thigh, and sternum. In total, six EMG signals and four 3D orientation signals were obtained via the Delsys Trigno Wireless system (Delsys Inc., Boston, MA, USA) and the sampling rates were 1777 and 74 Hz, respectively.

We also investigated the biomechanical response to the Myosuit under more physically demanding conditions by examining changes in gait patterns on an incline (Figure 1). Due to practical limitations of constructing, and controlling angled overground surfaces, we carried out the inclined gait sessions on M-Gait (Motekforce Link, Amsterdam, The Netherlands) which is a split-belt treadmill with dedicated 3D force plates and features pitch and speed control. Furthermore, we examined the effect of additional upper body load by providing the subjects with 10 kg weight vests. It has been recently suggested that in order to increase physical demand, weight vests need to exceed 10% of body mass [30]. Because no subject weighed more than 100 kg, we provided all subjects with a 10 kg weight vest. The joint angles were monitored identical to the aforementioned overground gait sessions, but the EMG sensor positions were slightly changed in order to evaluate VM.

In total, 11 healthy male subjects participated in this study. We initially recruited 7 subjects (174.4 ± 4.6 cm, 73.4 ± 12.5 kg, and 29.4 ± 4.8 years) to participate in the level overground gait trials. Upon completion of this study, we additionally recruited 4 subjects (175.7 ± 2.7 cm, 83.8 ± 6.1 kg, and 31.5 ± 3.9 years) to participate in the incline treadmill sessions. All subjects had no recent lower limb injuries (6 months), history of severe medical conditions, or cognitive issues that could interfere with the gait sessions. All gait sessions were conducted in the presence of a medical doctor and a “Myosuit operator” certified physiotherapist. The participants signed a written consent form and the research ethics of human experiments was ensured by conducting the gait sessions in accordance with the contents approved by the Institutional Review Board of Korea University (IRB No. 2021-0120-01).

#### 2.1.1. Myosuit

The Myosuit is a fully untethered and autonomous soft wearable robot, that weighs 5.6 kg, intended to assist with activities of daily life by operating like an external muscle. The user wears a backpack unit that contains two electric motors, a battery pack, and a control system. Both knee and hip extension are actively assisted via two cables, one on each leg, which extend from the motors and run across the back of the buttocks, thigh, and shank. Also, elastic bands are located on the front of the thigh which passively assists with hip and knee flexion. The Myosuit employs a position-based control approach to offer different operation modes. In the case of “Transparency” mode, the actuators are controlled such that there is no slack in the cables. Knee and hip extension torque is applied during the early stance phase for “Assist” mode [18,19].

#### 2.1.2. Level Overground Gait Sessions

The participants were asked to walk in a straight line along a level 20 m walkway for all overground gait conditions. During each gait trial, participants were instructed at the start line to “walk at a pace which feels comfortable and safe until the end of the walkway” and the data acquired from the first and last three gait cycles were excluded from the analysis. The overground gait sessions were comprised of a total of five gait conditions which include natural gait (NO-Myosuit) and “Transparency”, and “Assist” mode while wearing the Myosuit. The assist level was set at 1, 3, and 5 for “Assist” mode.

#### 2.1.3. Incline Treadmill Gait Sessions

In order to increase the overall task demand, we performed incline gait sessions on M-gait. The participants were asked to walk for 30 s on the treadmill set with 10° pitch at speeds of 2, 3, 4, and 5 km/h. The participants were given ample time to get accustomed to the treadmill settings and we started data acquisition once they gave a verbal cue that they felt comfortable. These conditions were performed with a 10 kg weight vest or “NO-Vest” in the presence and absence of the Myosuit. During the Myosuit treadmill gait trials, the mode was set to “Assist” mode assist level 5. The first and last 5 s from the acquired data were trimmed and used for further analysis to ensure that the target speed was reached and maintained.

### 2.2. Signal Processing

#### 2.2.1. EMG Pre-Processing

To compare muscle activation patterns during different gait conditions, we obtained an envelope function of the raw EMG signal by calculating the waveform length (WL) [31,32]. The raw EMG signal was first pre-processed via a band-pass filter (4th order Butterworth; 20 to 500 Hz) which was subsequently used to calculate the WL via Equation (1) shown below:

(1) $$EMG\;WL\left(n\right)=\sum_{i=n-N+2}^{n}\left|EMG\left(i\right)-EMG\left(i-1\right)\right|$$

where *N*, *n*, and EMG represent the window size, current sample, and raw signal, respectively.

Furthermore, $∆EMG\;WL_{Rel}$ was also obtained by calculating the normalized difference between the mean EMG WL within their respective gait-related task phases, as shown below:

(2) $$∆EMG\;WL_{Rel}=\frac{mean\;EMG\;WL_{condition}-mean\;EMG\;WL_{NO-Myosuit}}{mean\;EMG\;WL_{NO-Myosuit}}\times 100\%$$

where $mean\;EMG\;WL_{condition}$ and $mean\;EMG\;WL_{NO-Myosuit}$ represent the average EMG WL of the Myosuit worn and NO-Myosuit condition within the respective gait-related task phases, respectively.

#### 2.2.2. Joint Angle Estimation

The knee, hip, and ankle joint angles on the sagittal plane were estimated similar to that reported by Saito et al. [33]. We used the 3D orientation data provided by the IMU sensors, which utilize the acceleration, rotation, and earth magnetic field sensors, on the foot, shank, thigh, and sternum as shown in Figures S1 and S2. Because the IMU sensors were placed on the opposing sides of respective joints, the relative orientation of the two sensors could be used to estimate the joint angle [34].

The span of a specific joint during a gait-related task phase was assessed to evaluate any physical restrictions elicited by the Myosuit by calculating ∆JA via the equation shown below:

(3) $$∆JA=\max\left(joint\;angle\right)-\min\left(joint\;angle\right)$$

#### 2.2.3. Gait-Related Task Phase Segmentation

To assess the muscle activation patterns and ensuing movements, in accordance with their roles during gait, we propose to analyze the EMG patterns and joint angles within the gait-related task phases. It is well recognized that bipedal human gait is a repetition of three critical tasks, WA, SS, and LA, which need to be successfully executed in order to achieve forward progression while maintaining balance [27]. These tasks are carried out in separate well-defined phases of the gait cycle identifiable by ipsi- and contra-lateral heel contact and toe-off events [27].

**Figure 2:**
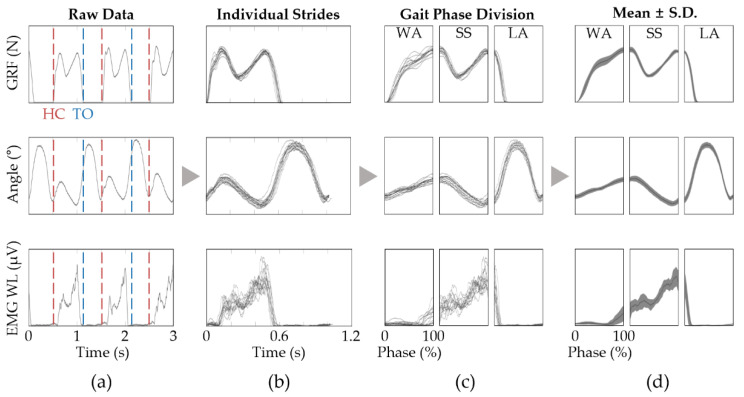
Signal processing overview. (**a**) The data are first synchronized and the heel contact (red vertical lines) and toe-off (blue vertical lines) positions are identified via applying a simple threshold method on the GRF; (**b**) The data are then segmented into individual strides; (**c**) We divide the data into WA, SS, and LA phase and then normalized into percentage of phase; (**d**) The mean and S.D. are plotted as a lines and shaded areas, respectively.

Initially, the joint angles and EMG WL data were synchronized with the ground reaction force (GRF) data which was obtained via Pedar-X (Novel GmbH, Munich, Germany) and M-gait for overground and treadmill gait sessions, respectively. Next, the heel contact and toe-off events for both limbs were identified by applying a simple threshold on the ipsi- and contra-lateral GRF signals. The timestamps of these gait events were utilized in order to accommodate the various sampling rates of the EMG, joint angles, and GRF signals. The synchronized data are then segmented into gait cycles (Figure 2a,b) which start and end with the current and subsequent ipsi-lateral heel contact, respectively. The gait cycle is further divided into the three non-overlapping task-related gait phases which are separated by contra-lateral toe-off and heel contact. The data are then normalized in time by the percent of the respective gait-related task phases (Figure 2c). The time traces within the respective phases are collectively represented as a single time trace using mean and standard deviation (S.D.) such that the gait patterns from different conditions could be overlayed and compared (Figure 2d). To assess whether any statistical significance could be asserted to the changes in EMG levels, the ensemble means of the EMG WL within the respective gait-related task phases while wearing the Myosuit were compared to that of NO-Myosuit using Student’s *t*-test. Furthermore, due to the small participant size, we used the Wilcoxon signed rank test to assess whether these changes were consistent throughout the participant group. All data processing was performed using a custom-built MATLAB (Mathworks Inc., Natick, MA, USA) code.

## 3. Results

### 3.1. Level Overground Gait Sessions

**Figure 3:**
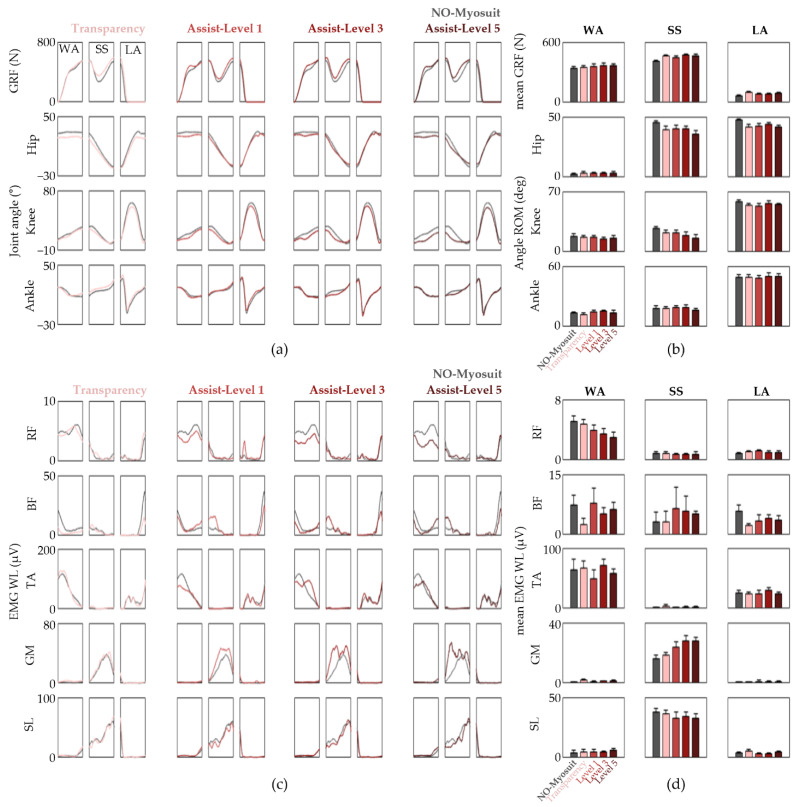
Gait patterns observed from overground gait sessions performed by subject 1. (**a**) The GRF and joint kinematics divided into the WA, SS, and LA phases; (**b**) The mean GRF is shown in the top row and the mean ∆JA for the lower limb joints within each phase are shown below; (**c**) EMG WL time traces divided into the gait-related task phases; (**d**) The average EMG WL within each phase. The data are represented as means ± S.D.

We acquired the GRF, joint kinematics, and EMG patterns from seven healthy adults while they walked along a straight 20 m overground path at a self-selected comfortable speed. The data were processed and presented such that the different Myosuit modes and assist levels could be compared with NO-Myosuit within the respective gait-related task phases (Figure 3). While some restrictions in knee joint angles are observed, specifically during the transition from WA to SS phase and mid-SS phase, it is clearly evident from Figure 3a,b that the overall joint kinematics are well preserved throughout the different conditions. This suggests that even with the maximum force profile (assist level 5), the Myosuit does not alter the subject’s natural lower limb movements during overground gait. However, at this point, it is yet to be elucidated whether active assistance is provided because one might expect similar results with a knee compression sleeve, which further highlights the importance of evaluating the underlying muscle activation patterns.

Thus, we analyzed the EMG WL patterns and the response to the different assist levels varied more than the joint kinematics (Figure 3c). Compared with NO-Myosuit, an assist-level-dependent reduction of the RF EMG WL was observed during the WA phase (Figure 3c,d). The ensemble averages of WA phase RF $∆EMG\;WL_{Rel}$ are −6.88, −22.19, −31.21, and −40.92% for “Transparency” mode and “Assist” mode set with assist level 1, 3, and 5, respectively (Figure 3d). There was also an assist-level-dependent increase of GM EMG WL in the SS phase which is understandable considering that plantar flexion favors GM over SL when the knee is extended [27,35], and that the assistance profile was designed to provide knee extension torque during the late WA and early SS phase. Collectively, our results suggest that the Myosuit indeed assists the user by lowering RF demand during the WA phase and the residual knee extension at the beginning of the SS phase elicits early GM activation without significantly altering the subject’s natural gait patterns. However, the extent to which EMG levels decreased was not as significant as we initially predicted. This may be due to the fact that overground natural gait among healthy subjects is significantly optimized.

### 3.2. Incline Treadmill Gait Sessions

We investigated whether the assistive effects of the Myosuit are more prominent when presented with a more challenging task by utilizing a pitch-control capable treadmill and a weighted vest. The four healthy subjects that participated in the incline treadmill gait sessions were asked to walk on a 10° incline at speeds of 2, 3, 4, and 5 km/h in the presence and absence of a 10 kg weighted vest. The EMG WL and joint kinematics from NO-Myosuit were compared to that of assist level five.

**Figure 4:**
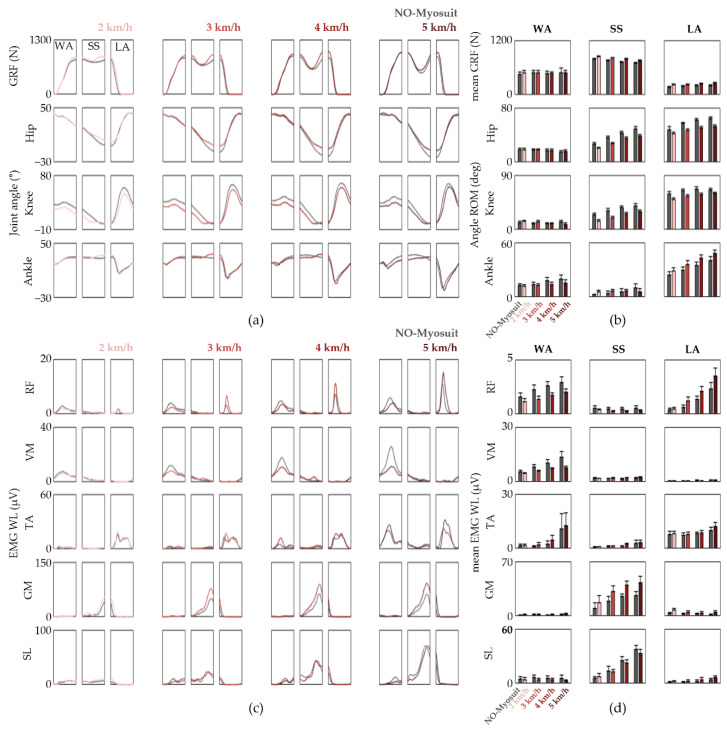
Gait patterns observed from incline treadmill gait sessions without additional trunk load performed by subject 1. (**a**) The GRF and joint kinematics divided into the WA, SS, and LA phases; (**b**) The mean GRF is shown in the top row and the mean ∆JA for the lower limb joints within each phase are shown below; (**c**) EMG WL time traces divided into the gait-related task phases; (**d**) The average EMG WL within each phase. The data are represented as means ± S.D.

**Figure 5:**
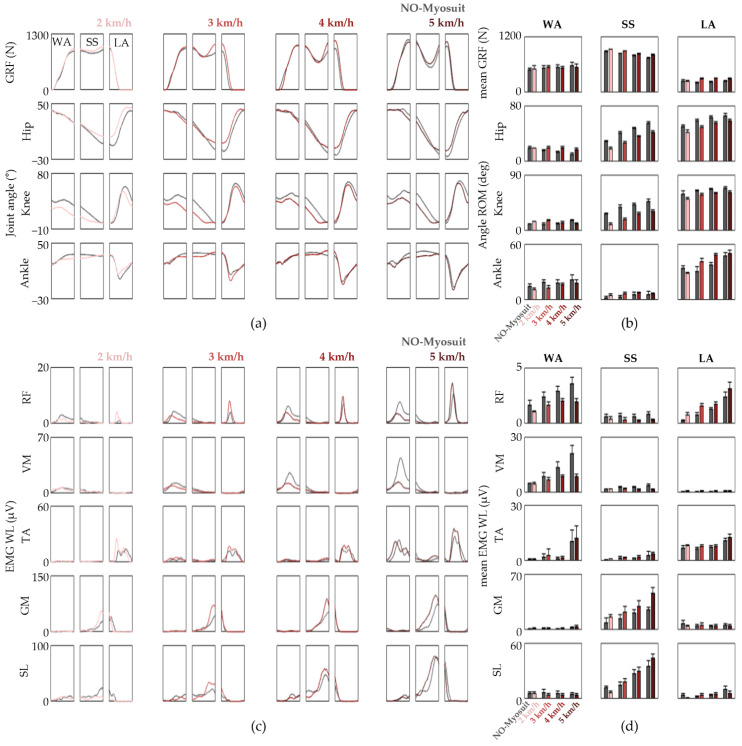
Gait patterns observed from incline treadmill gait sessions while wearing a 10 kg weighted vest performed by subject 1. (**a**) The GRF and joint kinematics divided into the WA, SS, and LA phases; (**b**) The mean GRF is shown in the top row and the mean ∆JA for the lower limb joints within each phase are shown below; (**c**) EMG WL time traces divided into the gait-related task phases; (**d**) The average EMG WL within each phase. The data are represented as means ± S.D.

In comparison to the previous overground gait sessions, the changes in lower limb biomechanics from the NO-Vest and 10 kg weighted vest conditions are indeed much more evident as shown in Figure 4 and Figure 5, respectively. With the assistance of the Myosuit, the knee flexion angles are reduced throughout the gait cycle, which was especially more apparent during the SS phase. For the NO-Vest conditions, reductions in the average knee ∆JA for 2, 3, 4, and 5 km/h was 10.27, 12.08, 10.20, and 10.15°, respectively (Figure 4b). Similar reduction levels were also observed when 10 kg vests were worn in order to provide additional trunk load (Figure 5a,b). We assert these changes as a response to the assistive extension torque applied by the Myosuit during the preceding WA phase. This postulation is supported by the EMG WL time traces which revealed a marked decrease in knee extensor activity during the WA phase (Figure 4c and Figure 5c). The evaluation of $∆EMG\;WL_{Rel}$ suggests a 26.90, 38.66, 34.19, and 31.28%, and 15.47, 25.35, 28.95, and 43.86% decrease in WA phase RF and VM EMG WL for 2, 3, 4, and 5 km/h, respectively, for NO-Vest conditions.

We also observed a slight increase in SL and GM EMG WL levels, similar to the overground gait sessions, during the SS phase which suggests additional plantar flexor demand. However, despite the aforementioned changes, the overall trends observed with both the joint angles and EMG WL time traces were not significantly altered much like what was observed with overground gait.

### 3.3. Group Analysis

**Figure 6:**
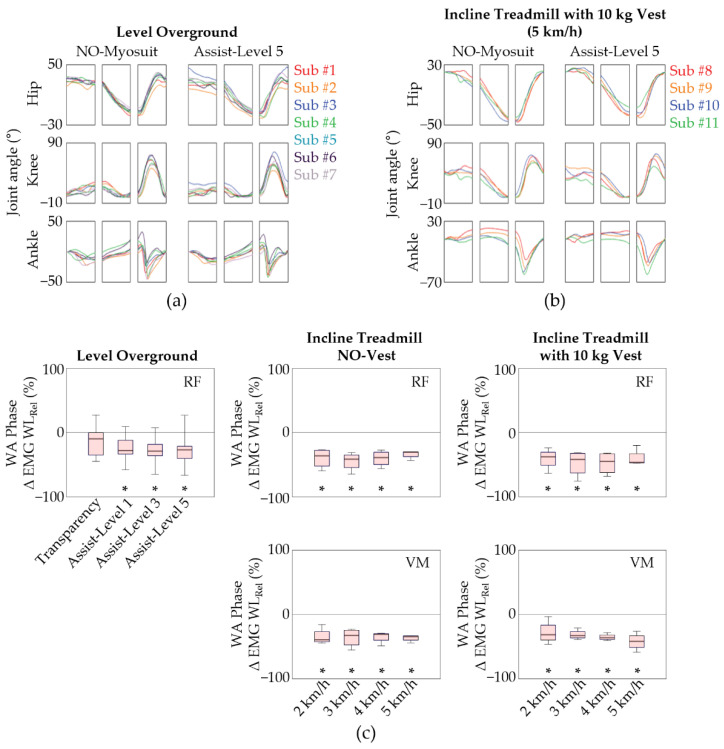
Group analysis of the biomechanical response to the Myosuit. The joint kinematic time traces of all participants for (**a**) level overground; (**b**) incline treadmill gait sessions; (**c**) Box plots show RF $∆EMG\;WL_{Rel}$ for level overground gait conditions and RF and VM $∆EMG\;WL_{Rel}$ for incline treadmill gait conditions. The data are represented as median (line within box), 25th and 75th percentiles (box), and minimum and maximum values (whiskers). * *p* < 0.05, assessed using the Wilcoxon signed rank test.

We then investigated the EMG WL patterns and joint angles of all participants in order to assess whether the biomechanical response to the Myosuit is consistent throughout the group (Figure 6). Much like what was observed with subject 1, the joint kinematic time traces from all participants showed some reductions in knee and hip range for level overground (Figure 6a and Tables S1–S7) and incline treadmill gait (Figure 6b and Tables S8–S15). The $∆EMG\;WL_{Rel}$ shown in Figure 6 were also in agreement with our assessment of subject 1. Collectively, the group analysis of the data acquired from both level and incline gait sessions suggests that the Myosuit reduces knee extensor demand without significantly impairing the users’ natural movements.

## 4. Discussion

In this study, we presented a thorough analysis of the biomechanical response of the lower limbs while wearing the Myosuit, which was designed to assist the user by providing knee and hip extension torque during the WA phase which helps facilitate bodyweight support and propulsive forces. The IMU and EMG sensors were placed such that the knee, hip, and ankle joint kinematics and muscle activation patterns could be evaluated during various gait conditions. For the level overground gait sessions, we compared the data obtained while walking at a comfortable speed wearing the Myosuit set with “Transparency” mode and “Assist” mode (level 1, 3, and 5) with that of the NO-Myosuit. Furthermore, in order to present more physically demanding conditions, treadmill gait sessions with 2, 3, 4, and 5 km/h and a 10° incline were also conducted.

We investigated the gait patterns by segmenting the signals into WA, SS, and LA phases to identify what muscles during which gait-related tasks were specifically assisted. The Student’s *t*-test evaluation of individual subjects (Tables S1–S15) and the Wilcoxon signed rank test of the entire group (Figure 6c) support that knee extensor EMG levels indeed decreased during the WA phase for both level and incline gait conditions. This agrees with the Myosuit’s intended design and these observations were present without any ensuing compensation patterns in the SS and LA phases. Furthermore, there were no obvious alterations in the lower limb joint kinematics and the EMG WL patterns of the evaluated muscles throughout the entire gait cycle. What was most noticeable was the reduction in knee extensor EMG levels during the WA phase for both level and incline gait conditions, which agrees with the Myosuit’s intended design. These observations were present without any significant alterations in lower limb joint kinematics and the EMG WL patterns of the evaluated muscles.

We would like to point out that while our EMG analysis indeed suggests significant reductions in knee extensor demand, the assistive effects of the Myosuit may not be confined to RF and VM because every muscle in the body was not evaluated. Thus, it is possible that the task demand for hip and/or core muscles were also reduced. Moreover, we believe that the limitations of estimating hip joint angles via the relative 3D orientation of the sternum and femur should be addressed because this method does not take lumbar flexion and extension into account. One might also raise the possibility that the reduced knee extensor demand during incline walking was caused by reduced step length. However, we assert no significant changes in step lengths considering that stride durations for the Myosuit-assisted and NO-Myosuit conditions (Figure S3) are similar for equal treadmill speed conditions. Finally, we did observe an increase in plantar flexor EMG WL levels in the SS phase. This may be due to the additional burden of supporting the Myosuit’s weight and/or limitations in knee flexion during the SS phase which has been shown to elicit higher plantar flexor demand [36].

Throughout this paper, we have reiterated that the Myosuit does not significantly alter the overall lower limb gait patterns for our experimental conditions. Emphasis was merited because natural gait is a complex physical activity that requires sequential execution of tasks via specific muscles or muscle groups. Additionally, training with non-physiological patterns, even if muscle demands or global metabolic costs are reduced, may form detrimental habits [37,38,39]. Thus, it is of utmost importance that the hallmark patterns such as the pre-activation of TA, RF, and BF and idle plantar flexors prior to the WA phase for limb stabilization and proper heel contact, increase of GM and SL EMG during the SS phase for forefoot rocker functions and toe-off, and TA activation during LA phase for foot clearance are preserved during level overground gait. Although walking uphill requires different surface negotiations and muscle execution strategies, the notion of preserving physiological patterns is still pertinent.

It has been reported that muscle mass decreases ~3–8% per decade after the age of 30 [40,41]. Thus, it is natural for one’s neuromusculoskeletal capacity, which has been previously defined as the physiological abilities of the neuromusculoskeletal system, to diminish with age [42,43]. However, the rate and extent depend on the muscle, and it is generally accepted that the larger proximal lower limb muscles show the most drastic changes [44,45]. Thus, compensation patterns or complete inability to perform certain physical tasks, such as gait, due to the diminished capacity of the proximal lower limb muscles can be commonly found among the elderly population. Although we observed reduced knee extensor demands during level and incline gait, the assistive effects may not directly translate to the aforementioned demographics due to the small sample size of our study. Thus, future EMG studies with participants that exhibit decreased hip and knee extension capacity such as the elderly, sedentary post-operation, and sarcopenia patients are warranted prior to the application in clinical settings.

## Data Availability

The data presented in this study are available on request from the corresponding author.

## Supplementary Materials

The following supporting information can be downloaded at: https://www.mdpi.com/article/10.3390/s22166127/s1, Figure S1: The EMG and IMU sensor placement positions; Figure S2: Schematic description of how the joint angles were estimated via 3D orientation data; Figure S3: The stride duration during incline treadmill gait sessions; Table S1: The average EMG WL within each phase observed from level overground gait sessions performed by subject 1; Table S2: The average EMG WL within each phase observed from level overground gait sessions performed by subject 2; Table S3: The average EMG WL within each phase observed from level overground gait sessions performed by subject 3; Table S4: The average EMG WL within each phase observed from level overground gait sessions performed by subject 4; Table S5: The average EMG WL within each phase observed from level overground gait sessions performed by subject 5; Table S6: The average EMG WL within each phase observed from level overground gait sessions performed by subject 6; Table S7: The average EMG WL within each phase observed from level overground gait sessions performed by subject 7; Table S8: The average EMG WL within each phase observed from incline treadmill gait sessions without additional trunk load performed by subject 8; Table S9: The average EMG WL within each phase observed from incline treadmill gait sessions without additional trunk load performed by subject 9; Table S10: The average EMG WL within each phase observed from incline treadmill gait sessions without additional trunk load performed by subject 10; Table S11: The average EMG WL within each phase observed from incline treadmill gait sessions without additional trunk load performed by subject 11; Table S12: The average EMG WL within each phase observed from incline treadmill gait sessions while wearing a 10 kg weighted vest performed by subject 8; Table S13: The average EMG WL within each phase observed from incline treadmill gait sessions while wearing a 10 kg weighted vest performed by subject 9; Table S14: The average EMG WL within each phase observed from incline treadmill gait sessions while wearing a 10 kg weighted vest performed by subject 10; Table S15: The average EMG WL within each phase observed from incline treadmill gait sessions while wearing a 10 kg weighted vest performed by subject 11.
