# Supplementary material for: Biomechanical Analysis Suggests Myosuit Reduces Knee Extensor Demand during Level and Incline Gait
Source: Sensors (Basel). 2022 Aug 16;22(16):6127. doi: 10.3390/s22166127 (PMC9413953; doi:10.3390/s22166127)
Supplement: Supplementary file 1 [file sensors-22-06127-s001.zip › sensors-1818624-supplementary.pdf]

sensors

MDPI

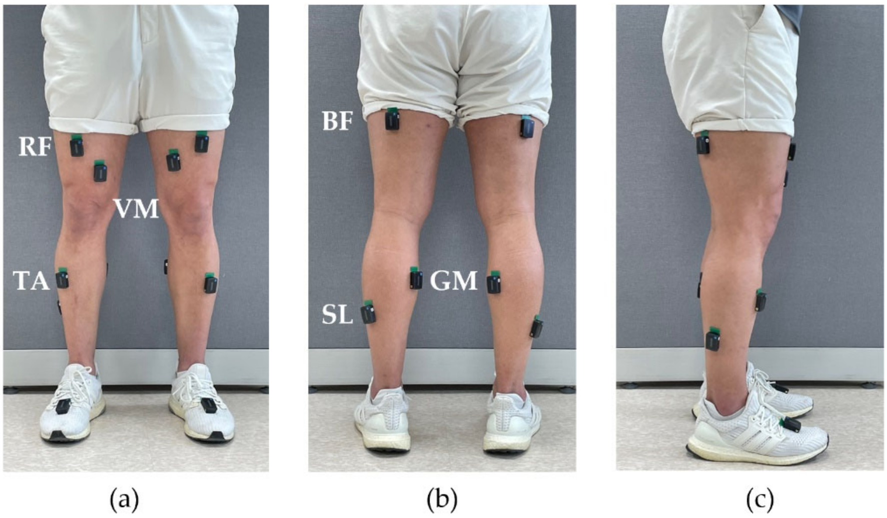


**Figure S1.** The EMG and IMU sensor placement positions. The EMG sensors were placed on the RF, BF, TA, GM, and SL for level overground gait sessions. During inclined treadmill gait sessions EMG signals from VM were monitored in place of BF. The (a) front, (b) back, and (c) side view is shown.

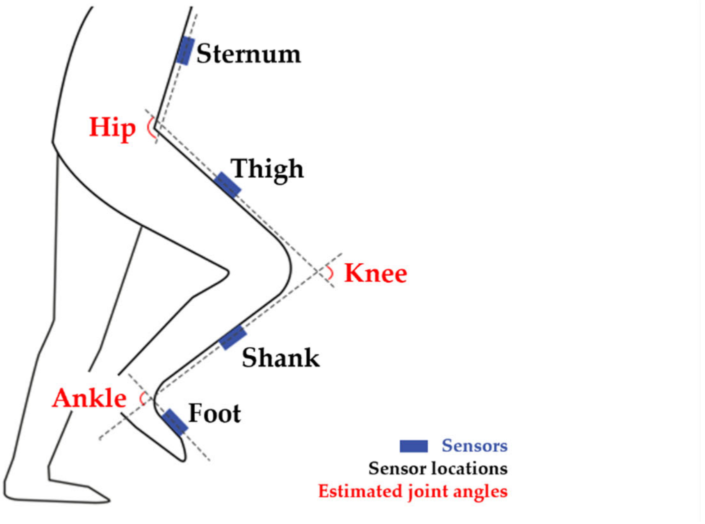


**Figure S2.** Schematic description of how the joint angles were estimated via 3D orientation data.

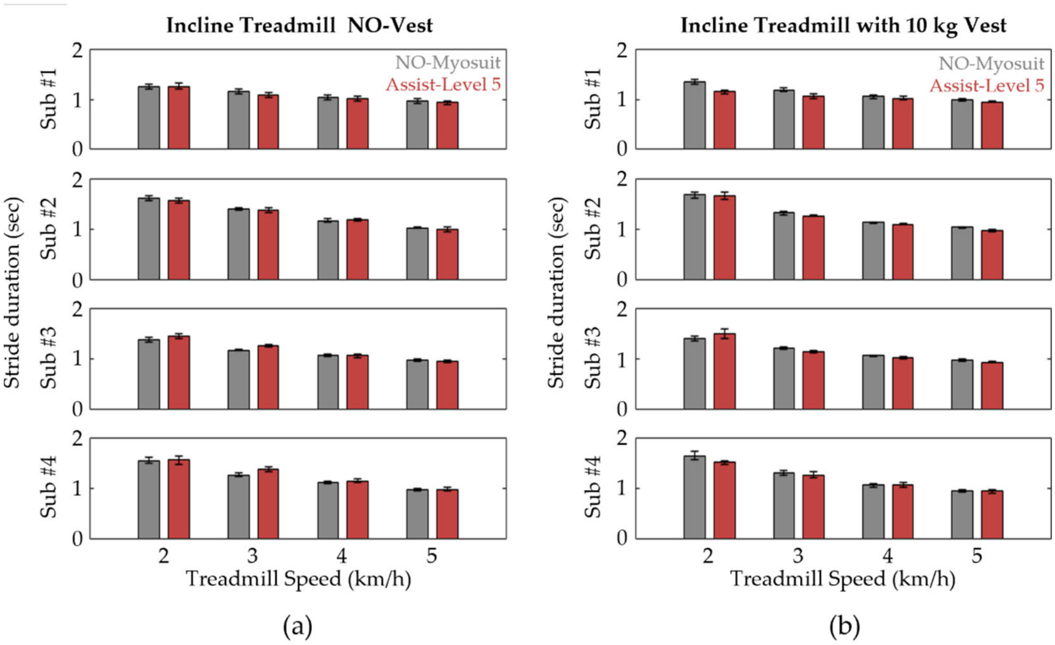


**Figure S3.** The stride duration during incline treadmill gait sessions. Data acquired from incline treadmill gait sessions under (a) NO-Vest and (b) with 10 kg weighted vest conditions. The data are represented as means ± S.D.

**Table S1.** The average EMG WL within each phase observed from level overground gait sessions performed by subject 1.

| | | NO-Myosuit | Transparency | Assist-Level 1 | Assist-Level 3 | Assist-Level 5 |
|---|---|---|---|---|---|---|
| WA | RF | 5.1±0.7 | 4.7±0.6 | 4.0±0.6* | 3.5±0.6* | 3.0±0.7* |
| | BF | 7.4±2.4 | 2.6±1.4* | 7.7±3.9 | 5.1±1.7* | 6.2±2.0 |
| | TA | 64.6±16.5 | 66.3±11.8 | 48.7±14.6* | 70.8±10.4 | 58.2±7.1 |
| | GM | 0.8±0.2 | 2.1±0.6* | 1.0±0.5 | 1.2±0.5* | 1.3±0.5 |
| | SL | 4.3±2.2 | 5.0±1.8 | 4.9±2.0 | 4.4±1.1 | 6.0±2.0 |
| SS | RF | 0.9±0.2 | 0.9±0.2 | 0.7±0.1 | 0.8±0.1 | 0.8±0.3 |
| | BF | 3.3±2.4 | 3.1±2.7 | 6.5±5.2 | 5.9±3.7 | 5.2±0.6* |
| | TA | 2.1±0.7 | 3.6±2.3* | 1.6±0.2* | 2.4±1.1 | 2.4±1.1 |
| | GM | 16.1±2.2 | 18.5±1.9* | 23.7±3.4* | 27.7±3.6* | 27.7±2.4* |
| | SL | 38.0±2.9 | 36.6±2.6 | 32.8±5.0* | 34.3±3.9* | 32.6±3.9 |
| LA | RF | 0.9±0.2 | 1.1±0.2* | 1.2±0.2* | 1.0±0.2 | 0.9±0.3 |
| | BF | 5.9±1.6 | 2.3±0.5* | 3.5±1.5* | 4.1±0.9* | 3.6±1.2* |
| | TA | 26.4±4.3 | 24.9±2.9 | 25.0±4.7 | 30.1±3.8* | 24.6±2.6 |
| | GM | 0.7±0.2 | 1.0±0.2* | 1.2±0.8 | 1.1±0.4* | 1.1±0.2* |
| | SL | 3.8±1.1 | 5.4±1.5* | 3.3±1.0 | 3.2±1.1 | 4.5±1.0 |

**Table S2.** The average EMG WL within each phase observed from level overground gait sessions performed by subject 2.

| | | NO-Myosuit | Transparency | Assist-Level1 | Assist-Level3 | Assist-Level5 |
|---|---|---|---|---|---|---|
| WA | RF | 2.1±0.5 | 1.9±0.3 | 0.9±0.1* | 0.7±0.1* | 0.7±0.1* |
| | BF | 7.8±2.3 | 2.9±0.9* | 3.8±1.1* | 4.2±1.2* | 4.1±1.6* |
| | TA | 5.6±1.8 | 3.5±2.8* | 6.8±2.2 | 5.4±2.1 | 5.7±2.6 |
| | GM | 2.5±0.9 | 3.5±2.0* | 3.1±1.5 | 4.0±1.4* | 4.2±1.5* |
| | SL | 2.5±1.0 | 7.9±2.5* | 4.3±1.2* | 4.9±1.0* | 5.3±0.8* |
| SS | RF | 0.9±0.2 | 0.8±0.1 | 0.3±0.1* | 0.4±0.1* | 0.4±0.1* |
| | BF | 0.5±0.6 | 0.4±0.1 | 0.2±0.1 | 0.3±0.1 | 0.3±0.1 |
| | TA | 0.6±0.4 | 1.1±0.6* | 1.0±0.2* | 1.1±0.2* | 1.1±0.1* |
| | GM | 7.6±1.0 | 7.8±1.5 | 9.3±2.1* | 9.3±1.6* | 9.7±1.6* |
| | SL | 18.1±2.3 | 20.9±2.6* | 15.3±1.8* | 14.6±1.4* | 14.2±1.2* |
| LA | RF | 0.5±0.1 | 0.5±0.1 | 0.2±0.0* | 0.2±0.0* | 0.2±0.0* |
| | BF | 2.2±0.5 | 2.6±0.7* | 1.9±0.5 | 1.7±0.3* | 2.1±0.4 |
| | TA | 6.1±1.1 | 10.1±1.4* | 11.1±1.6* | 11.9±1.6* | 10.6±2.2* |
| | GM | 0.9±0.3 | 0.7±0.2* | 0.7±0.3* | 0.7±0.4 | 0.9±0.3 |
| | SL | 3.4±1.3 | 1.5±0.6* | 2.3±0.8* | 2.3±0.7* | 2.3±0.7* |

**Table S3.** The average EMG WL within each phase observed from level overground gait sessions performed by subject 3.

| | | NO-Myosuit | Transparency | Assist-Level1 | Assist-Level3 | Assist-Level5 |
|---|---|---|---|---|---|---|
| WA | RF | 2.5±0.3 | 2.0±0.2* | 2.3±0.3* | 2.0±0.3* | 1.8±0.4* |
| | BF | 1.8±0.4 | 2.1±0.3 | 1.8±0.4 | 1.5±0.5* | 1.4±0.3* |
| | TA | 48.4±6.4 | 52.3±6.5 | 47.8±7.6 | 44.8±5.2 | 39.8±9.3* |
| | GM | 0.5±0.1 | 0.5±0.1 | 0.5±0.1 | 0.5±0.1 | 0.4±0.1 |
| | SL | 1.7±0.3 | 4.0±1.2* | 6.4±3.0* | 2.8±0.8* | 2.9±1.8* |
| SS | RF | 0.5±0.1 | 0.7±0.1* | 0.7±0.1* | 0.6±0.1 | 0.6±0.2 |
| | BF | 0.3±0.1 | 0.5±0.2* | 0.3±0.1 | 0.3±0.0 | 0.3±0.1 |
| | TA | 1.1±0.6 | 1.8±0.9* | 3.1±1.6* | 2.9±0.9* | 2.1±1.2* |
| | GM | 12.9±1.6 | 13.2±1.5 | 9.9±1.4* | 8.9±1.6* | 7.6±2.9* |
| | SL | 14.2±1.9 | 14.5±1.4 | 10.7±1.5* | 9.5±0.9* | 8.9±1.5* |
| LA | RF | 0.6±0.1 | 0.4±0.1* | 0.6±0.1 | 0.4±0.1* | 0.3±0.1* |
| | BF | 1.9±0.1 | 1.3±0.1* | 1.3±0.1* | 1.2±0.1* | 1.3±0.2* |
| | TA | 12.2±1.7 | 11.4±1.8 | 14.9±3.7* | 10.9±0.9* | 9.8±1.3* |
| | GM | 1.9±0.3 | 3.8±0.8* | 3.7±0.8* | 3.7±0.7* | 3.6±0.9* |
| | SL | 3.1±0.6 | 5.8±0.8* | 4.2±0.8* | 4.0±0.8* | 3.3±0.5 |

**Table S4.** The average EMG WL within each phase observed from level overground gait sessions performed by subject 4.

| | | NO-Myosuit | Transparency | Assist-Level1 | Assist-Level3 | Assist-Level5 |
|---|---|---|---|---|---|---|
| WA | RF | 2.4±0.5 | 3.0±0.6* | 2.6±0.5 | 2.6±0.9 | 3.0±0.7* |
| | BF | 5.7±1.7 | 6.5±1.5 | 9.6±2.2* | 10.1±2.0* | 9.6±3.7* |
| | TA | 54.1±7.0 | 34.0±5.8* | 32.6±3.7* | 40.2±9.0* | 49.8±5.6 |
| | GM | 2.2±2.7 | 6.1±4.3* | 1.4±0.6 | 1.5±1.1 | 4.1±3.6 |
| | SL | 6.9±3.1 | 4.3±1.5* | 8.2±5.7 | 6.8±1.7 | 6.1±2.1 |
| SS | RF | 4.9±1.7 | 7.7±0.7* | 4.7±0.4 | 5.7±1.4 | 8.1±1.5* |
| | BF | 0.6±0.2 | 0.6±0.3 | 0.6±0.4 | 1.2±0.5* | 2.1±1.0* |
| | TA | 2.9±1.1 | 2.9±1.5 | 2.5±0.5 | 2.6±0.9 | 2.8±1.1 |
| | GM | 28.2±2.0 | 37.0±3.9* | 27.1±2.1 | 30.5±3.6 | 34.6±5.5* |
| | SL | 30.9±2.6 | 30.1±1.9 | 30.8±2.2 | 29.8±3.2 | 30.2±2.2 |
| LA | RF | 0.9±0.3 | 0.7±0.2* | 0.7±0.2 | 0.6±0.2* | 0.5±0.2* |
| | BF | 2.0±0.4 | 2.1±0.3 | 2.7±0.8 | 2.6±0.7* | 2.0±0.4 |
| | TA | 15.0±1.8 | 12.5±1.1* | 12.3±0.9* | 12.5±1.6* | 13.6±1.4* |
| | GM | 4.3±1.0 | 5.9±2.2* | 3.8±1.0 | 4.5±0.9 | 7.1±4.4 |
| | SL | 27.4±3.8 | 12.4±2.4* | 7.5±1.4* | 9.4±2.3* | 10.0±2.4* |

**Table S5.** The average EMG WL within each phase observed from level overground gait sessions performed by subject 5.

| | | NO-Myosuit | Transparency | Assist-Level1 | Assist-Level3 | Assist-Level5 |
|---|---|---|---|---|---|---|
| WA | RF | 2.1±0.3 | 2.2±0.3 | 1.5±0.2* | 1.3±0.2* | 1.6±0.3* |
| | BF | 3.4±0.4 | 3.6±0.8 | 3.9±0.7 | 3.8±0.7 | 3.5±0.6 |
| | TA | 14.4±3.7 | 22.8±5.2* | 23.9±3.6* | 22.5±6.0* | 26.8±4.1* |
| | GM | 1.6±0.5 | 1.4±0.1 | 0.9±0.3* | 1.2±0.4* | 1.8±1.4 |
| | SL | 6.4±1.2 | 6.6±2.6 | 1.9±0.5* | 2.4±0.5* | 2.6±0.7* |
| SS | RF | 0.4±0.1 | 0.6±0.1* | 0.7±0.1* | 1.8±1.5* | 7.1±1.4* |
| | BF | 0.6±0.1 | 0.9±0.2* | 1.6±0.2* | 1.7±0.3* | 1.6±0.5* |
| | TA | 0.9±0.7 | 1.9±2.1 | 2.2±1.8 | 6.1±5.6* | 8.0±5.1* |
| | GM | 24.6±3.1 | 26.1±3.0 | 18.1±2.0* | 14.7±2.9* | 8.9±3.4* |
| | SL | 18.6±2.0 | 25.1±3.4* | 19.2±2.5 | 20.9±1.9* | 18.8±4.0 |
| LA | RF | 0.4±0.1 | 0.4±0.1 | 0.4±0.1 | 0.3±0.1 | 0.4±0.2 |
| | BF | 0.5±0.2 | 0.6±0.1 | 0.9±0.3* | 1.7±0.5* | 1.6±0.4* |
| | TA | 11.8±1.3 | 19.3±2.3* | 8.2±0.8* | 8.5±0.5* | 9.4±1.0* |
| | GM | 1.0±0.3 | 1.3±0.1* | 5.4±1.1* | 4.8±1.2* | 6.4±1.2* |
| | SL | 2.6±0.5 | 3.8±0.7* | 6.3±1.1* | 7.3±1.3* | 11.1±2.8* |

**Table S6.** The average EMG WL within each phase observed from level overground gait sessions performed by subject 6.

| | | NO-Myosuit | Transparency | Assist-Level1 | Assist-Level3 | Assist-Level5 |
|---|---|---|---|---|---|---|
| WA | RF | 2.8±0.4 | 1.5±0.4* | 1.8±0.3* | 2.3±0.6 | 2.2±0.4* |
| | BF | 3.6±0.6 | 2.4±0.8* | 1.9±0.7* | 2.2±0.5* | 2.1±0.2* |
| | TA | 33.4±1.8 | 28.2±8.9* | 29.1±6.1* | 32.2±4.8 | 33.6±7.6 |
| | GM | 0.7±0.2 | 0.9±0.4 | 0.8±0.2 | 1.2±0.6 | 0.9±0.2 |
| | SL | 3.1±1.1 | 2.8±0.6 | 3.4±0.9 | 4.1±0.9 | 4.2±0.7 |
| SS | RF | 0.7±0.2 | 1.1±0.3* | 0.5±0.2* | 1.0±0.3* | 1.1±0.5 |
| | BF | 0.6±0.1 | 1.6±0.5* | 0.3±0.1* | 1.6±0.3* | 1.4±0.2* |
| | TA | 2.2±2.1 | 4.9±4.8* | 2.4±2.1 | 3.1±3.4 | 3.5±3.3 |
| | GM | 11.5±1.3 | 13.7±4.4* | 15.3±1.7* | 15.7±2.7* | 14.3±2.6* |
| | SL | 18.0±1.4 | 16.1±1.9* | 18.2±1.3 | 18.5±1.9 | 18.3±1.7 |
| LA | RF | 0.9±0.2 | 1.0±0.3 | 0.7±0.2 | 0.9±0.2 | 0.8±0.2 |
| | BF | 1.8±0.3 | 1.4±0.4* | 1.7±0.3 | 1.7±0.2 | 1.5±0.1* |
| | TA | 10.6±1.5 | 6.3±2.2* | 9.4±1.0 | 8.9±1.1* | 9.6±1.3 |
| | GM | 3.2±1.2 | 5.1±1.5* | 2.4±0.7 | 3.9±1.3 | 3.8±1.5 |
| | SL | 3.9±0.8 | 6.5±1.3* | 3.8±1.0 | 5.2±1.2* | 4.7±1.0 |

**Table S7.** The average EMG WL within each phase observed from level overground gait sessions performed by subject 7.

| | | NO-Myosuit | Transparency | Assist-Level1 | Assist-Level3 | Assist-Level5 |
|---|---|---|---|---|---|---|
| WA | RF | 1.2±0.1 | 0.7±0.2* | 0.9±0.2* | 0.9±0.2* | 0.8±0.2* |
| | BF | 0.3±0.1 | 1.3±0.2* | 2.0±0.3* | 2.0±0.2* | 5.6±1.0* |
| | TA | 12.5±3.1 | 14.8±3.5 | 13.3±3.1 | 18.5±2.6 | 16.4±4.7 |
| | GM | 0.9±0.5 | 0.7±0.3 | 0.8±0.5 | 0.7±0.2 | 1.7±1.5 |
| | SL | 2.4±0.6 | 1.5±0.4 | 1.9±0.6 | 1.8±0.5 | 1.9±0.6 |
| SS | RF | 0.7±0.1 | 0.4±0.1* | 0.4±0.1* | 0.5±0.1* | 0.4±0.1* |
| | BF | 0.3±0.0 | 0.5±0.1* | 0.4±0.1* | 0.4±0.1* | 0.6±0.2* |
| | TA | 1.0±0.4 | 2.1±1.3* | 1.0±0.3 | 1.6±0.8 | 4.0±1.9* |
| | GM | 17.5±1.2 | 23.2±2.5* | 26.0±3.3* | 28.5±3.2* | 27.0±3.4* |
| | SL | 11.1±1.0 | 9.5±1.1 | 10.4±1.1 | 6.3±0.7* | 6.7±0.8* |
| LA | RF | 0.4±0.1 | 0.3±0.1 | 0.4±0.1 | 0.4±0.1 | 0.3±0.1 |
| | BF | 0.8±0.2 | 0.4±0.1 | 0.5±0.1 | 0.4±0.1 | 0.5±0.1 |
| | TA | 6.8±0.4 | 7.9±0.5* | 6.7±0.5 | 7.1±0.5 | 6.3±0.9 |
| | GM | 0.5±0.0 | 3.0±1.2* | 1.5±0.8* | 4.1±1.0* | 3.5±1.6* |
| | SL | 1.8±0.2 | 3.1±0.8* | 2.7±0.4* | 2.7±0.5* | 2.3±0.6 |

**Table S8.** The average EMG WL within each phase observed from incline treadmill gait sessions without additional trunk load performed by subject 8.

| | | 2 km/h | | 3 km/h | | 4 km/h | | 5 km/h | |
|---|---|---|---|---|---|---|---|---|---|
| | | NO-Myosuit | Assist-Level5 | NO-Myosuit | Assist-Level5 | NO-Myosuit | Assist-Level5 | NO-Myosuit | Assist-Level5 |
| **WA** | **RF** | 1.6±0.4 | 1.2±0.3* | 2.2±0.4 | 1.4±0.3* | 2.6±0.3 | 1.7±0.2* | 2.9±0.5 | 2.0±0.3* |
| | **VM** | 5.4±0.9 | 4.6±0.7* | 8.0±1.4 | 5.9±0.7* | 10.3±1.9 | 7.3±0.6* | 13.6±3.1 | 7.7±0.9* |
| | **TA** | 1.4±1.2 | 1.5±0.8 | 1.1±0.5 | 1.9±1.5* | 2.4±1.8 | 4.6±2.5* | 11.0±8.4 | 12.7±6.7 |
| | **GM** | 1.3±0.6 | 1.3±0.9 | 1.6±0.9 | 1.4±1.1 | 1.3±0.5 | 1.6±1.4 | 1.8±0.6 | 2.3±1.3 |
| | **SL** | 5.2±1.9 | 4.3±2.4 | 6.6±2.4 | 4.1±1.7* | 5.7±2.5 | 3.8±2.0* | 6.0±2.9 | 2.9±1.2* |
| **SS** | **RF** | 0.5±0.2 | 0.4±0.1* | 0.5±0.1 | 0.3±0.1* | 0.5±0.2 | 0.3±0.1* | 0.5±0.2 | 0.3±0.1* |
| | **VM** | 1.8±0.5 | 1.8±0.3 | 1.8±0.3 | 2.0±0.3* | 1.7±0.4 | 2.3±0.3* | 2.0±0.7 | 2.3±0.4* |
| | **TA** | 0.8±0.5 | 0.9±0.3 | 1.0±0.4 | 1.5±0.3* | 1.4±0.3 | 2.3±0.5* | 2.7±1.9 | 3.5±1.3 |
| | **GM** | 10.2±6.7 | 16.6±9.4* | 19.4±6.1 | 31.8±6.9* | 25.3±3.2 | 39.7±5.5* | 26.6±4.5 | 42.4±8.3* |
| | **SL** | 5.8±2.0 | 7.6±3.5 | 13.9±4.7 | 13.1±2.6 | 25.5±4.5 | 22.1±4.2* | 37.5±4.4 | 32.7±4.9* |
| **LA** | **RF** | 0.4±0.1 | 0.5±0.2 | 0.7±0.2 | 1.2±0.4* | 1.4±0.3 | 2.1±0.5* | 2.3±0.6 | 3.5±0.8* |
| | **VM** | 0.5±0.1 | 0.6±0.1 | 0.7±0.2 | 0.7±0.1 | 0.7±0.2 | 0.7±0.2 | 1.0±0.2 | 0.8±0.2* |
| | **TA** | 7.8±1.9 | 8.3±1.2 | 7.5±0.9 | 7.8±1.4 | 8.0±1.0 | 8.6±1.2 | 10.0±1.8 | 12.0±2.2* |
| | **GM** | 3.4±1.2 | 7.2±2.5* | 2.3±1.3 | 4.9±1.8* | 2.1±1.2 | 3.8±2.0* | 1.7±0.8 | 5.1±1.8* |
| | **SL** | 1.3±0.6 | 2.4±1.1* | 1.7±0.7 | 2.6±1.5* | 2.6±1.3 | 4.2±2.3* | 3.8±2.0 | 6.4±2.2* |

**Table S9.** The average EMG WL within each phase observed from incline treadmill gait sessions without additional trunk load performed by subject 9.

| | | 2 km/h | | 3 km/h | | 4 km/h | | 5 km/h | |
|---|---|---|---|---|---|---|---|---|---|
| | | NO-Myosuit | Assist-Level5 | NO-Myosuit | Assist-Level5 | NO-Myosuit | Assist-Level5 | NO-Myosuit | Assist-Level5 |
| WA | RF | 2.3±0.4 | 1.6±0.3* | 2.5±0.4 | 1.7±0.4* | 3.1±0.5 | 2.2±0.4* | 3.3±0.4 | 2.3±0.3* |
| | VM | 22.3±4.2 | 13.9±2.7* | 28.3±3.8 | 17.2±4.4* | 31.5±3.0 | 21.6±3.1* | 35.9±5.2 | 24.1±3.2* |
| | TA | 16.8±13.7 | 27.5±18.4 | 17.9±8.6 | 26.6±4.4* | 40.0±20.0 | 71.5±21.7* | 57.6±21.4 | 65.4±22.0 |
| | GM | 1.7±0.5 | 2.5±1.6 | 1.8±0.6 | 3.3±1.6 | 1.7±0.6 | 2.1±0.8 | 2.2±0.5 | 2.4±1.1 |
| | SL | 8.3±4.0 | 9.7±5.1 | 10.0±4.0 | 15.7±5.9 | 6.2±3.4 | 5.0±2.2 | 5.6±2.8 | 6.6±3.4 |
| SS | RF | 0.9±0.3 | 1.7±0.4* | 0.8±0.2 | 0.7±0.2 | 0.8±0.1 | 0.9±0.2 | 0.7±0.1 | 0.5±0.1* |
| | VM | 5.5±1.5 | 7.4±1.9* | 5.3±2.0 | 4.3±0.9 | 5.7±1.2 | 5.3±1.6 | 5.3±0.9 | 3.6±0.7* |
| | TA | 7.0±8.5 | 13.4±4.3* | 4.9±2.5 | 12.6±5.8* | 9.2±7.6 | 23.4±9.2* | 12.1±8.6 | 20.1±8.9* |
| | GM | 19.0±6.3 | 16.5±7.2 | 28.6±4.1 | 31.1±6.5 | 32.0±3.5 | 40.6±5.1* | 39.5±3.8 | 46.4±4.8* |
| | SL | 13.2±3.2 | 16.5±2.8* | 22.8±4.0 | 25.7±5.6 | 30.4±3.7 | 33.6±3.3* | 38.8±4.2 | 38.2±4.4 |
| LA | RF | 0.3±0.1 | 0.2±0.0 | 0.4±0.1 | 0.2±0.0* | 1.2±0.5 | 1.0±0.1 | 2.9±0.6 | 3.3±0.7* |
| | VM | 0.3±0.1 | 0.5±0.1* | 0.5±0.1 | 0.4±0.1 | 0.6±0.2 | 0.4±0.1* | 1.3±0.4 | 0.5±0.2* |
| | TA | 14.2±1.0 | 20.8±2.3* | 17.1±1.7 | 23.2±2.8* | 20.8±1.8 | 25.8±3.7* | 29.0±2.5 | 33.0±3.2* |
| | GM | 6.8±1.6 | 12.8±1.7* | 3.0±0.9 | 9.6±2.1* | 2.3±0.5 | 5.8±0.9* | 1.9±0.5 | 3.9±1.1* |
| | SL | 5.2±1.3 | 10.2±2.3* | 4.8±1.6 | 12.5±0.9* | 5.0±1.9 | 9.0±2.8* | 4.7±1.3 | 7.0±1.5* |

**Table S10.** The average EMG WL within each phase observed from incline treadmill gait sessions without additional trunk load performed by subject 10.

| | | 2 km/h | | 3 km/h | | 4 km/h | | 5 km/h | |
|---|---|---|---|---|---|---|---|---|---|
| | | NO-Myosuit | Assist-Level5 | NO-Myosuit | Assist-Level5 | NO-Myosuit | Assist-Level5 | NO-Myosuit | Assist-Level5 |
| WA | RF | 2.4±0.4 | 1.0±0.2* | 3.2±0.5 | 1.1±0.2* | 3.4±0.5 | 1.5±0.2* | 3.5±0.5 | 2.0±0.4* |
| | VM | 11.4±1.5 | 6.3±1.2* | 16.9±2.0 | 7.6±0.9* | 18.7±2.9 | 9.7±1.0* | 19.9±4.0 | 12.8±2.0* |
| | TA | 6.0±5.5 | 3.5±1.2 | 5.7±2.4 | 9.6±6.2 | 13.1±6.3 | 22.0±8.5* | 28.3±9.5 | 28.9±6.4 |
| | GM | 1.0±0.7 | 2.1±1.4* | 0.8±0.5 | 0.6±0.2 | 0.6±0.2 | 0.6±0.2 | 1.1±1.0 | 0.7±0.2 |
| | SL | 4.2±2.5 | 9.2±4.5* | 3.7±3.7 | 2.8±2.6 | 3.6±2.0 | 2.8±1.8 | 3.3±1.0 | 3.5±1.4 |
| SS | RF | 1.1±0.2 | 0.9±0.2* | 1.0±0.3 | 0.8±0.1* | 0.9±0.2 | 0.8±0.1 | 0.7±0.1 | 1.1±0.2* |
| | VM | 3.1±0.6 | 3.3±0.8 | 3.3±0.9 | 3.1±0.6 | 3.2±0.6 | 2.6±0.6* | 2.8±0.4 | 2.2±0.6* |
| | TA | 2.2±1.8 | 3.9±2.0* | 1.9±0.5 | 4.0±2.4* | 3.4±0.9 | 5.8±3.0* | 4.3±0.5 | 5.5±3.8 |
| | GM | 27.7±6.7 | 19.8±7.4* | 28.8±5.4 | 30.7±5.6 | 36.2±4.8 | 42.0±4.9* | 50.6±3.3 | 47.8±6.0 |
| | SL | 12.7±7.8 | 17.2±6.7 | 21.6±5.4 | 20.0±6.9 | 40.8±7.5 | 31.7±5.8* | 52.0±6.4 | 40.7±4.8* |
| LA | RF | 0.4±0.1 | 0.2±0.1* | 0.6±0.2 | 0.4±0.1* | 0.7±0.1 | 0.8±0.1* | 1.1±0.1 | 1.1±0.2 |
| | VM | 0.3±0.1 | 0.5±0.2* | 0.4±0.1 | 0.3±0.1 | 0.4±0.1 | 0.4±0.1 | 0.4±0.1 | 0.4±0.1 |
| | TA | 9.1±1.7 | 7.2±1.3* | 9.2±0.9 | 7.5±1.0* | 9.6±1.6 | 9.3±1.0 | 14.3±1.0 | 12.1±1.9* |
| | GM | 5.1±1.6 | 11.5±1.9* | 3.3±1.4 | 10.3±2.2* | 2.5±0.9 | 8.4±1.6* | 3.5±1.8 | 9.4±1.5* |
| | SL | 6.7±1.7 | 11.7±2.6* | 6.7±1.5 | 13.7±3.0* | 6.9±1.5 | 12.6±2.6* | 8.3±1.5 | 15.0±3.1* |

**Table S11.** The average EMG WL within each phase observed from incline treadmill gait sessions without additional trunk load performed by subject 11.

| | | 2 km/h | | 3 km/h | | 4 km/h | | 5 km/h | |
|---|---|---|---|---|---|---|---|---|---|
| | | NO-Myosuit | Assist-Level5 | NO-Myosuit | Assist-Level5 | NO-Myosuit | Assist-Level5 | NO-Myosuit | Assist-Level5 |
| WA | RF | 2.1±0.5 | 1.2±0.3* | 2.5±0.4 | 1.4±0.3* | 2.3±0.4 | 1.3±0.3* | 2.5±0.3 | 1.7±0.2* |
| | VM | 4.0±0.7 | 2.4±0.7* | 4.2±0.5 | 3.2±0.6* | 4.7±0.9 | 3.3±0.4* | 6.5±1.5 | 4.4±0.7* |
| | TA | 2.9±2.0 | 4.1±1.9 | 4.0±1.4 | 6.4±2.6* | 8.3±2.7 | 10.1±3.8 | 12.7±3.6 | 17.2±3.7* |
| | GM | 0.7±0.4 | 1.3±1.3 | 0.6±0.4 | 0.6±0.4 | 0.5±0.2 | 0.6±0.5 | 0.8±0.4 | 0.6±0.2 |
| | SL | 2.3±1.4 | 3.1±2.2 | 1.7±1.2 | 1.8±1.3 | 1.5±0.8 | 1.6±1.0 | 1.8±0.8 | 1.7±0.4 |
| SS | RF | 0.8±0.2 | 0.9±0.2 | 0.8±0.2 | 1.0±0.2* | 0.7±0.2 | 0.8±0.2 | 0.8±0.2 | 0.9±0.2 |
| | VM | 1.3±0.3 | 1.1±0.2 | 1.3±0.4 | 1.5±0.3* | 1.3±0.2 | 1.4±0.3 | 2.1±0.5 | 1.6±0.3* |
| | TA | 1.6±0.7 | 3.7±2.6* | 2.5±0.4 | 3.6±1.5* | 3.5±0.4 | 5.1±1.7* | 5.2±0.7 | 5.6±0.8* |
| | GM | 11.9±4.1 | 10.5±4.3 | 17.7±3.6 | 13.9±2.9* | 24.7±1.7 | 24.0±4.9 | 30.4±2.6 | 32.5±3.9* |
| | SL | 8.8±1.8 | 9.0±2.6 | 12.1±2.2 | 11.3±2.9 | 17.5±1.6 | 18.2±2.9 | 22.3±2.1 | 19.3±2.8* |
| LA | RF | 0.4±0.1 | 0.3±0.1* | 0.5±0.2 | 0.3±0.1* | 0.9±0.2 | 0.5±0.1* | 1.6±0.3 | 0.7±0.2* |
| | VM | 1.5±0.5 | 0.4±0.1* | 2.0±0.5 | 0.3±0.1* | 2.0±0.4 | 0.3±0.1* | 2.7±0.6 | 0.4±0.1* |
| | TA | 4.4±0.5 | 5.8±0.7* | 5.5±0.7 | 6.6±0.6* | 6.6±0.7 | 7.7±0.8* | 8.3±1.0 | 8.5±0.7 |
| | GM | 3.8±1.3 | 6.3±2.0* | 2.4±1.4 | 6.5±1.7* | 2.7±1.0 | 5.2±2.2* | 2.0±0.7 | 5.2±1.3* |
| | SL | 3.5±1.3 | 6.8±2.3* | 2.9±1.2 | 7.4±1.5* | 3.2±1.1 | 5.7±2.2* | 2.9±0.8 | 5.1±0.7* |

**Table S12.** The average EMG WL within each phase observed from incline treadmill gait sessions while wearing a 10 kg weighted vest performed by subject 8.

| | | 2 km/h | | 3 km/h | | 4 km/h | | 5 km/h | |
|---|---|---|---|---|---|---|---|---|---|
| | | NO-Myosuit | Assist-Level5 | NO-Myosuit | Assist-Level5 | NO-Myosuit | Assist-Level5 | NO-Myosuit | Assist-Level5 |
| WA | RF | 1.7±0.4 | 1.0±0.1* | 2.4±0.5 | 1.6±0.3* | 2.9±0.4 | 2.0±0.3* | 3.6±0.6 | 1.9±0.3* |
| | VM | 4.9±0.2 | 4.7±0.7 | 8.8±1.9 | 7.0±1.0* | 13.5±2.9 | 8.5±0.9* | 20.9±4.5 | 8.7±1.3* |
| | TA | 0.7±0.4 | 0.8±0.3 | 2.0±1.9 | 2.9±3.5 | 1.3±0.5 | 1.5±0.8 | 10.3±6.0 | 12.3±6.2 |
| | GM | 0.9±0.2 | 1.5±0.6 | 1.5±0.9 | 1.3±1.1 | 1.6±0.4 | 1.7±0.5 | 2.5±0.9 | 3.6±1.6* |
| | SL | 5.4±2.5 | 5.8±1.7 | 6.4±3.7 | 3.7±2.2* | 6.1±2.3 | 4.4±1.4* | 4.5±2.4 | 4.2±1.3 |
| SS | RF | 0.7±0.2 | 0.4±0.2 | 0.7±0.3 | 0.3±0.2* | 0.6±0.2 | 0.3±0.1* | 0.8±0.2 | 0.3±0.1* |
| | VM | 2.1±0.1 | 2.0±0.1 | 2.8±0.6 | 2.0±0.4* | 2.9±0.6 | 1.7±0.4* | 3.6±1.1 | 1.7±0.4* |
| | TA | 0.8±0.1 | 0.9±0.1 | 1.5±0.9 | 1.6±0.5 | 1.3±0.4 | 2.0±0.6* | 3.0±2.2 | 3.8±0.8 |
| | GM | 9.0±6.1 | 16.0±3.0 | 14.3±5.2 | 22.6±7.2* | 20.9±4.8 | 29.4±6.7* | 24.9±3.4 | 46.0±6.6* |
| | SL | 11.7±2.2 | 6.6±2.0* | 14.3±3.3 | 17.7±3.9* | 26.8±4.7 | 29.5±4.3 | 35.1±6.3 | 43.4±4.8* |
| LA | RF | 0.3±0.1 | 0.8±0.2 | 0.8±0.2 | 1.6±0.3* | 1.3±0.2 | 1.7±0.2* | 2.4±0.5 | 3.1±0.6* |
| | VM | 0.6±0.1 | 0.8±0.1 | 0.5±0.1 | 0.8±0.1* | 0.5±0.1 | 0.8±0.2* | 0.8±0.3 | 0.9±0.2 |
| | TA | 6.6±1.4 | 8.0±0.5 | 6.4±1.1 | 7.5±1.1* | 7.1±0.9 | 7.5±1.0 | 10.8±1.5 | 12.7±1.6* |
| | GM | 7.3±4.8 | 4.7±1.1 | 4.6±1.9 | 6.1±2.4* | 4.5±0.9 | 5.1±1.4 | 5.8±1.5 | 5.0±1.4 |
| | SL | 3.8±2.0 | 1.2±0.4 | 2.3±0.8 | 4.0±1.9* | 3.8±1.1 | 5.2±1.7* | 10.4±3.2 | 6.0±2.2* |

**Table S13.** The average EMG WL within each phase observed from incline treadmill gait sessions while wearing a 10 kg weighted vest performed by subject 9.

| | | 2 km/h | | 3 km/h | | 4 km/h | | 5 km/h | |
|---|---|---|---|---|---|---|---|---|---|
| | | NO-Myosuit | Assist-Level5 | NO-Myosuit | Assist-Level5 | NO-Myosuit | Assist-Level5 | NO-Myosuit | Assist-Level5 |
| WA | RF | 1.7±0.2 | 1.3±0.3* | 2.6±0.3 | 1.7±0.2* | 3.3±0.4 | 2.2±0.3* | 4.1±0.5 | 2.2±0.3* |
| | VM | 17.2±1.8 | 11.5±3.0* | 26.4±4.2 | 18.1±4.1* | 32.6±3.5 | 21.2±3.3* | 40.8±4.3 | 23.1±2.5* |
| | TA | 8.4±4.7 | 15.4±6.8* | 16.9±11.3 | 22.3±6.2 | 44.8±11.3 | 52.6±23.8 | 69.9±13.8 | 62.9±14.3 |
| | GM | 2.0±0.6 | 3.0±1.3* | 1.8±0.6 | 2.2±1.1 | 1.3±0.3 | 1.8±0.7* | 1.7±0.4 | 1.9±0.8 |
| | SL | 11.0±3.4 | 9.6±4.1 | 8.8±4.0 | 11.8±2.7 | 3.9±1.6 | 6.6±4.3 | 3.4±1.1 | 5.9±2.2* |
| SS | RF | 1.0±0.3 | 1.1±0.2 | 0.9±0.2 | 0.8±0.1* | 1.1±0.2 | 0.7±0.2* | 1.4±0.2 | 0.4±0.1* |
| | VM | 5.7±2.1 | 5.4±0.9 | 6.3±1.5 | 4.4±0.8* | 8.3±1.7 | 4.8±1.2* | 11.8±2.1 | 3.5±0.7* |
| | TA | 2.9±1.5 | 10.5±4.5* | 3.8±2.2 | 6.2±3.7 | 10.0±7.9 | 12.0±6.0 | 11.9±6.6 | 21.5±8.7* |
| | GM | 18.5±7.0 | 25.2±6.0* | 24.7±3.4 | 39.3±3.7* | 29.1±3.4 | 42.0±3.9* | 36.0±3.4 | 45.5±4.8* |
| | SL | 17.6±3.4 | 17.9±2.6 | 21.5±3.3 | 22.4±2.7 | 28.4±3.0 | 28.0±3.8 | 35.7±3.9 | 33.5±3.6 |
| LA | RF | 0.2±0.1 | 0.2±0.1* | 0.6±0.2 | 0.4±0.1* | 1.2±0.3 | 1.1±0.2 | 2.4±0.6 | 3.6±0.7* |
| | VM | 0.3±0.1 | 0.4±0.1 | 0.4±0.1 | 0.4±0.1 | 0.5±0.1 | 0.6±0.1* | 0.6±0.1 | 0.4±0.1* |
| | TA | 11.9±0.7 | 17.8±3.0* | 14.2±1.6 | 21.5±2.5* | 20.0±1.3 | 26.1±1.3* | 25.0±1.6 | 34.3±3.4* |
| | GM | 9.3±2.0 | 13.2±2.4* | 7.6±1.1 | 7.1±1.9 | 6.4±1.2 | 7.0±1.3 | 5.8±1.2 | 7.0±1.2* |
| | SL | 7.7±2.0 | 9.2±2.6 | 8.1±1.5 | 7.4±2.1 | 9.5±1.5 | 8.5±1.6 | 10.2±2.1 | 10.0±1.7 |

**Table S14.** The average EMG WL within each phase observed from incline treadmill gait sessions while wearing a 10 kg weighted vest performed by subject 10.

| | | 2 km/h | | 3 km/h | | 4 km/h | | 5 km/h | |
|---|---|---|---|---|---|---|---|---|---|
| | | NO-Myosuit | Assist-Level5 | NO-Myosuit | Assist-Level5 | NO-Myosuit | Assist-Level5 | NO-Myosuit | Assist-Level5 |
| WA | RF | 2.3±0.5 | 0.9±0.2* | 3.0±0.3 | 1.5±0.3* | 3.8±0.5 | 1.7±0.2* | 3.9±0.8 | 2.0±0.4* |
| | VM | 10.6±1.5 | 5.7±1.5* | 15.0±1.5 | 9.2±1.0* | 19.7±2.9 | 11.8±1.0* | 22.8±3.0 | 13.7±2.0* |
| | TA | 6.6±10.7 | 6.0±3.2 | 4.7±3.1 | 8.6±3.7* | 15.0±7.2 | 14.8±6.6 | 31.3±13.5 | 27.6±5.6 |
| | GM | 1.0±0.4 | 2.3±3.0 | 0.8±0.4 | 0.4±0.2* | 0.9±0.9 | 0.6±0.2 | 1.5±1.3 | 0.7±0.2* |
| | SL | 5.2±3.5 | 5.2±3.0 | 4.2±2.9 | 2.7±3.8 | 2.7±1.9 | 2.6±1.5 | 5.0±2.8 | 2.7±0.8* |
| SS | RF | 1.0±0.3 | 0.9±0.3 | 0.8±0.2 | 0.8±0.2 | 0.8±0.1 | 0.7±0.1 | 0.8±0.2 | 1.2±0.2* |
| | VM | 2.8±0.7 | 3.3±1.0 | 2.6±0.4 | 3.0±0.6 | 2.7±0.6 | 2.9±0.8 | 3.4±0.9 | 2.4±0.8* |
| | TA | 2.4±2.7 | 5.8±4.0* | 2.0±0.7 | 3.8±2.2* | 3.8±1.0 | 4.5±3.0 | 5.6±1.2 | 5.3±3.1 |
| | GM | 21.1±7.2 | 12.7±5.8* | 32.8±6.3 | 23.5±4.9* | 39.7±3.1 | 36.9±5.3 | 51.6±5.2 | 42.6±5.8* |
| | SL | 10.1±3.2 | 11.7±6.6 | 24.1±5.9 | 11.2±3.9* | 41.0±5.2 | 25.7±9.2* | 58.8±6.6 | 35.1±4.9* |
| LA | RF | 0.4±0.1 | 0.3±0.1* | 0.6±0.1 | 0.7±0.1* | 0.8±0.1 | 0.8±0.1 | 1.1±0.2 | 1.1±0.2 |
| | VM | 0.3±0.1 | 0.4±0.1* | 0.3±0.1 | 0.4±0.1 | 0.5±0.1 | 0.4±0.1* | 0.5±0.1 | 0.4±0.1* |
| | TA | 6.7±1.3 | 7.7±1.4 | 8.8±1.1 | 7.7±0.7* | 10.7±1.6 | 10.8±1.7 | 14.9±2.3 | 12.7±1.7* |
| | GM | 3.0±1.4 | 16.0±2.0* | 2.0±0.8 | 13.0±2.4* | 2.1±0.9 | 13.7±2.2* | 1.8±0.5 | 14.7±2.3* |
| | SL | 5.4±1.4 | 14.0±3.4* | 6.0±1.5 | 12.0±3.9* | 6.6±2.3 | 15.8±2.3* | 6.7±1.9 | 20.2±2.0* |

**Table S15.** The average EMG WL within each phase observed from incline treadmill gait sessions while wearing a 10 kg weighted vest performed by subject 11.

| | | 2 km/h | | 3 km/h | | 4 km/h | | 5 km/h | |
|---|---|---|---|---|---|---|---|---|---|
| | | **NO-Myosuit** | **Assist-Level5** | **NO-Myosuit** | **Assist-Level5** | **NO-Myosuit** | **Assist-Level5** | **NO-Myosuit** | **Assist-Level5** |
| **WA** | **RF** | 2.2±0.5 | 1.4±0.4* | 5.8±2.0 | 1.4±0.2* | 4.6±2.2 | 1.5±0.2* | 2.3±0.6 | 1.9±0.5 |
| | **VM** | 4.2±0.7 | 2.9±1.1* | 4.7±1.1 | 3.1±0.5 | 4.6±2.0 | 3.3±0.5 | 5.9±2.4 | 4.4±0.9 |
| | **TA** | 3.9±3.0 | 3.9±4.0 | 3.6±1.8 | 5.1±2.3 | 7.6±2.9 | 9.2±3.1 | 11.7±0.8 | 14.1±3.5* |
| | **GM** | 0.7±0.5 | 0.8±0.4 | 0.8±0.6 | 0.5±0.5 | 0.5±0.2 | 0.5±0.3 | 1.3±0.6 | 1.7±1.0 |
| | **SL** | 1.9±1.7 | 2.3±1.4 | 1.7±1.3 | 1.5±1.2 | 1.3±0.9 | 1.2±0.5 | 1.4±0.2 | 1.9±0.7* |
| **SS** | **RF** | 1.0±0.3 | 1.1±0.4 | 0.9±0.2 | 1.0±0.2 | 1.2±0.8 | 0.7±0.2 | 1.2±0.1 | 0.8±0.2* |
| | **VM** | 1.9±0.3 | 1.3±0.5* | 2.2±0.3 | 1.4±0.3* | 2.2±0.4 | 1.3±0.3* | 2.5±0.7 | 1.6±0.4 |
| | **TA** | 1.6±1.1 | 2.4±1.5 | 2.1±0.6 | 3.2±0.7* | 3.7±0.7 | 5.0±0.6* | 5.0±0.8 | 8.7±1.5* |
| | **GM** | 7.2±3.8 | 10.9±4.5* | 17.1±3.8 | 20.3±4.0 | 22.1±1.8 | 26.6±5.3* | 31.6±1.2 | 39.1±4.2* |
| | **SL** | 5.8±3.0 | 9.9±3.3* | 13.3±2.0 | 16.3±4.9 | 15.4±2.3 | 18.7±2.8* | 23.1±0.4 | 25.0±2.9* |
| **LA** | **RF** | 0.5±0.2 | 0.4±0.1 | 1.1±0.6 | 0.4±0.1 | 2.5±0.9 | 0.8±0.2* | 1.4±0.4 | 1.1±0.3 |
| | **VM** | 1.6±0.7 | 0.3±0.1* | 2.0±0.6 | 0.3±0.0* | 3.4±1.1 | 0.4±0.1* | 7.6±1.1 | 0.4±0.1* |
| | **TA** | 4.5±0.9 | 3.9±0.4 | 4.9±0.4 | 4.6±0.5 | 5.7±1.0 | 6.1±0.7 | 7.6±0.4 | 8.4±0.8* |
| | **GM** | 6.6±2.6 | 4.7±1.8 | 3.8±1.3 | 4.5±1.0 | 4.3±1.0 | 4.1±1.4 | 4.2±0.9 | 3.8±1.2 |
| | **SL** | 4.6±1.7 | 4.8±1.9 | 3.7±0.9 | 5.6±1.4* | 4.6±1.2 | 4.4±1.0 | 4.4±0.7 | 5.3±1.0 |

*, $p < 0.05$, assessed using Student's t-test for all tables.
